# Comprehensive characterization of RNA cargo of extracellular vesicles in breast cancer patients undergoing neoadjuvant chemotherapy

**DOI:** 10.3389/fonc.2022.1005812

**Published:** 2022-10-26

**Authors:** Lilite Sadovska, Pawel Zayakin, Kristaps Eglītis, Edgars Endzeliņš, Ilze Radoviča-Spalviņa, Elīza Avotiņa, Jānis Auders, Laura Keiša, Inta Liepniece-Karele, Mārcis Leja, Jānis Eglītis, Aija Linē

**Affiliations:** ^1^ Cancer Biomarker group, Latvian Biomedical Research and Study Centre, Riga, Latvia; ^2^ Latvian Oncology Center, Riga Eastern Clinical University Hospital, Riga, Latvia; ^3^ Genera Ltd., Riga, Latvia; ^4^ Institute of Clinical and Preventive Medicine, University of Latvia, Riga, Latvia; ^5^ Department of Pathology, Riga Stradins University, Riga, Latvia; ^6^ University of Latvia, Faculty of Medicine, University of Latvia, Riga, Latvia

**Keywords:** extracellular vesicles, RNA biotypes, snoRNA, breast cancer, neoadjuvant chemotherapy, RNA sequencing, liquid biopsy, prognostic biomarker

## Abstract

Extracellular vesicles (EVs) are g7aining increased attention as carriers of cancer-derived molecules for liquid biopsies. Here, we studied the dynamics of EV levels in the plasma of breast cancer (BC) patients undergoing neoadjuvant chemotherapy (NAC) and explored the relevance of their RNA cargo for the prediction of patients’ response to the therapy. EVs were isolated from serial blood samples collected at the time of diagnosis, at the end of NAC, and 7 days, 6, and 12 months after the surgery from 32 patients with locally advanced BC, and 30 cancer-free healthy controls (HCs) and quantified by nanoparticle tracking analysis. The pre-treatment levels of EVs in BC patients were higher than in HCs, significantly increased during the NAC and surgery, and decreased to the levels found in HCs 6 months after surgery, thus showing that a substantial fraction of plasma EVs in BC patients are produced due to the disease processes and treatment. RNA sequencing analysis revealed that the changes in the EV levels were associated with the alterations in the proportions of various RNA biotypes in EVs. To search for RNA biomarkers that predict response to the NAC, patients were dichotomized as responders and non-responders based on Miller-Payne grades and differential expression analyses were carried out between responders and non-responders, and HCs. This resulted in the identification of 6 miRNAs, 4 lncRNAs, and 1 snoRNA that had significantly higher levels in EVs from non-responders than responders at the time of diagnosis and throughout the NAC, and significantly lower levels in HCs, thus representing biomarkers for the prediction of response to NAC at the time of diagnosis. In addition, we found 14 RNAs representing piRNA, miRNA, lncRNA, snoRNA, and snRNA biotypes that were induced by NAC in non-responders and 2 snoRNAs and 1 piRNA that were induced by NAC in patients with early disease progression, thus warranting further functional studies on their role in chemoresistance and metastasis.

## Introduction

Neoadjuvant chemotherapy (NAC) is increasingly used in the management of patients with locally advanced breast cancer (BC) to render inoperable tumors operable, reduce the extent of surgery, and prevent the spreading of metastatic cells (1, 2). Moreover, pre-operative chemotherapy allows to assess the drug sensitivity of the tumor. Response to NAC provides important prognostic information and correlates with long-term outcomes to some extent (3). Patients with triple-negative BC (TNBC), HER2-positive or high-grade hormone receptor (HR)-positive tumors that achieve pathological complete response (pCR) to NAC have significantly longer disease-free and overall survival, whereas the pCR is not well correlated to the outcome in patients with low grade, slowly proliferating HR-positive tumors (2). Furthermore, the majority of patients do not achieve pCR to NAC and the prognostic significance of partial responses is less clear. Several staging systems for assessing prognosis after NAC have been developed, including the Miller-Payne (4), residual cancer burden (5), and Neo-Bioscore (6) grading systems, though they require tumor tissue specimens for the histological examination and typically are applied once the NAC is completed and their prediction accuracy varies in different subtypes of BC. Furthermore, increasing evidence suggests that in a subset of patients chemotherapeutic drugs may lead to a pathological complete response of the primary tumor but promote metastasis in the NAC setting (7). Hence, a non-invasive blood-based assay that would allow the prediction of response before or during the NAC and identification of patients who are at risk of the disease progression after NAC would be of great benefit for the management of patients with locally advanced BC.

Liquid biopsies are samples of body fluids that are used for the analysis of circulating tumor cells (CTCs) or cancer-derived molecules such as cell-free tumor DNA or RNA (8, 9). They hold great promise for the diagnosis, prediction, or monitoring of response to treatment and early detection of recurrence in BC patients. Recently, extracellular vesicles (EVs) have emerged as alternative carriers of cancer-derived molecules in liquid biopsies. EVs contain a wide variety of RNA biotypes - fragments of mRNAs and long non-coding RNAs (lncRNAs), microRNAs (miRNAs), piwi-interacting RNAs (piRNAs), small nuclear RNAs (snRNAs), small nucleolar RNAs (snoRNAs), tRNA-derived small RNAs (tRFs and tRNA halves), circular RNAs (circRNAs), vault-RNAs, Y-RNAs, etc. (10, 11). Although several sorting mechanisms may lead to enrichment or depletion of EVs with some RNAs, overall, the composition of EV-RNA resembles that of their parental cell (12). These findings have led to the idea that the analysis of EV-RNA content could inform about the presence, molecular profile, and behavior of cancer.

This study aimed to evaluate the relevance of EV RNA cargo for the management of patients with locally advanced BC. We performed EV RNA sequencing analysis in serial blood samples collected at various time points from 32 BC patients undergooing NAC and 30 cancer-free females. In addition, full transcriptome libraries were prepared from BC and normal breast tissues from 10 patients. Patients were dichotomized as responders and non-responders based on Miller-Payne grades. To identify cancer-derived EV-RNA biomarkers that predict the response to NAC at the time of diagnosis, we searched for EV-RNAs that were differentially expressed between responders and non-responders and had significantly higher levels in BC patients compared to cancer-free controls. In addition, we searched for EV-RNAs that were induced by NAC, absent or low in controls and higher in non-responders than responders, thus suggesting that the induction of their expression may functionally contribute to drug resistance and cancer cell survival.

## Materials and methods

### Study population and sample collection

BC patients were recruited between June 2019 and October 2020 at Riga East University Hospital and followed-up 18 months after the surgery. Inclusion criteria: previously untreated invasive primary BC diagnosed by core needle biopsy, stage II to III at diagnosis, age 18-78 years, prescribed NAC. Exclusion criteria: blood transfusion in the last six months, another oncological disease. The blood samples were collected at specified time points – at the time of diagnosis, at the end of NAC, 7 days after the surgery, and 6, 12, and 18 months after the surgery. Blood samples were collected in EDTA-coated tubes and processed at room temperature within 2 hours. Plasma samples were centrifuged twice at 3000*g* for 10 min, aliquoted, and stored at -80°C. BC and adjacent normal tissue specimens were macroscopically dissected by a histopathologist during surgery and stored in RNALater (Applied Biosystems, USA) at -20°C till processing. Plasma samples from 30 cancer-free age-matched women were obtained from the Latvian Genome Database.

The NAC regimens contained Doxorubicin, Docetaxel, Cyclophosphamide, Paclitaxel, 5FU, and Epirubicin. The patients were dichotomized based on the Miller-Payne grades: patients with grades 1 to 3 were classified as non-responders and patients with grades 4-5 as responders. The characteristics of the study population are provided in **Table 1**.

**Table 1:** Clinical characteristics of the study population.

Characteristic	All BC patients	Responders	Non-Responders	*P* value
Sample size (n)	35	12	20	*na*
Age mean, years	51.2	50.9	53.4	
Age range, years	34-77	35-74	36-77	
Tumor grade				
Grade 2	23	8	12	0.706
Grade 3	12	4	8	
TNM stage				
T1 N1-3 M0	1	0	1	0.854
T2 N1-3 M0	14	5	8	
T3 N1-3 M0	18	7	9	
T4 N1-3 M0	2	0	2	
Estrogen receptor				
Positive	23	9	11	0.258
Negative	12	3	9	
Progesterone receptor				
Positive	17	5	9	0.854
Negative	18	7	11	
HER2 overexpression				
0	6	3	3	0.926
1	14	4	9	
2	4	0	3	
3	11	5	5	
TNBC				
Yes	8	3	3	0.483
No	27	9	17	
E-cadherin				
Positive	24	8	13	0.923
Negative	11	4	7	
Proliferation Index (Ki-67)				
≤14%	6	1	5	0.242
> 14%	29	11	15	
NAC regimens				
Dox, Pac	6	0	6	*na*
Dox, Cycl	22	8	11	
Epi, Herc, Doc	1	0	1	
Dox, Cycl, 5FU	1	0	1	
Epi, Cycl, Doc	1	1	0	
Dox, Cycl, Doc, Tras	2	2	0	
Dox, Cycl, Pac	1	1	0	
Number of chemotherapy courses				
4	3	1	1	*na*
6	6	1	5	
7	1	0	1	
8	21	9	10	
12	1	0	1	
Response to chemotherapy (Miller-Payne)				
1-3	20	0	20	*na*
4-5	12	12	0	
Disease progression within 18 months post-Op				
Yes	11	4	7	0.923
No	23	8	13	

NAC, Neoadjuvant chemotherapy; TNBC, Triple-negative breast cancer; Dox, Doxorubicin; Pac, Paclitaxel; Cycl, Cyclophosphamide; Epi, Epirubicin; Herc, Herceptin; Doc, Docetaxel; Tras, Trastazumab; 5FU, Fluorouracil; na, not applicable.

The study was conducted according to the Declaration of Helsinki. The specimens were collected after the patients’ informed written consent was obtained. The samples were stored in the Latvian Genome Database. The biobanking procedures have been approved by the Latvian Central Medical Ethics Committee (first approval No. 2007 A-7, renewed approvals No.1/19-04-05 and No. 01-29.1.2/6407) and the use of clinical samples for this study was approved by the Committee of Biomedical Ethics of Riga East University Hospital and the Latvian Central Medical Ethics Committee (approval No. 1839).

### Isolation and characterization of extracellular vesicles

EVs were isolated from 1 ml of plasma, using size exclusion chromatography (SEC). SEC columns were prepared from 10 ml of Sepharose CL2B (Cytiva, USA) in TELOS SPE columns (Kinesis, USA). Plasma samples were loaded on the columns and gravity-eluted with PBS-DEPC, and the eluate was collected in 15 sequential 500 μl fractions. Fractions were measured with Zetasizer Nano ZS (Malvern, UK) and fractions containing particles larger than 35 nm, were collected and concentrated to 100 μl using Amicon Ultra 3 kDa centrifugal filters (Merck Millipore, Germany). To check the purity and quality, EVs from 4 patients and controls were analyzed by transmission electron microscopy (TEM) as described before (13). All samples were routinely measured by nanoparticle tracking analysis (NTA) using the NanoSight NS500 instrument (Malvern, UK). For the measurement, the EVs were diluted 1000-4000 times in filtered PBS. For each sample, five 60-second videos were recorded with the following settings: 25C, 0.944–0.948 cP, slider shutter 1259, slider gain 366, and camera level 11. Data analysis was performed using NanoSight NTA Software v3.1 Build 3.1.54.

### Western blot analysis

EVs were heated for 5 min at 95°C with reducing Laemmli buffer and amounts corresponding to 100 μL plasma were loaded per lane of a 10% SDS-PAGE gel. After separation, the proteins were transferred to nitrocellulose membranes, which were subsequently blocked using 10% (w/v) fat-free milk. Membranes were incubated with primary antibodies against TSG101 (Abcam, #ab15011, 1:1000 dilution), Calnexin (Abcam, #ab22595, 1:2000 dilution) and PDCD6IP/ALIX (Santa Cruz Biotechnology, #sc-166952, 1:1000 dilution) overnight at +4˚C. After washing in TBST, membranes were incubated for 1h at room temperature with anti-rabbit IgG, F(ab’)2-HRP (Santa Cruz Biotechnology, #sc-3837, 1:2000 dilution), goat anti-mouse m-IgG BP-HRP (Santa Cruz Biotechnology, #sc-516102, 1:2000 dilution), or HRP-conjugated antibody against CD63 (Novus Biologicals, #NBP2-34779H, 1:2000 dilution). After washing in TBST, immunoreactive bands were visualized using Amersham™ ECL Select™ Western Blotting Detection Reagent kit (GE HealthCare Lifesciences) and pictures were taken using a Nikon d610 dSLR body (Nikon) with Sigma 35mm f/1.4 DG HSM Art lens (Sigma).

### RNA extraction

Before RNA extraction, the EV samples were treated with 1 mg/ml proteinase K (Thermo Fisher Scientific, USA) for 30 min at + 37˚C. Proteinase was inactivated by heating the sample at + 65˚C for 10 minutes, and then the samples were treated with 10 ng/μl RNAse A (Thermo Fisher Scientific, USA) for 15 minutes at +37˚C. Immediately after that, EV samples were lysed by adding 5 volumes of QIAzol Lysis reagent and EV-RNA was extracted using miRNeasy Micro Kit (Qiagen, USA) according to the manufacturer’s protocol. Briefly, 1 ml of chloroform was added to the EV lysate, incubated at room temperature for 10 minutes, and centrifuged for 15 minutes at 12000*g* at +4˚C. The aqueous phase was transferred to a fresh tube and 1.5 vol of 100% ethanol was added, and the mixture was loaded onto the MinElute Spin column. Columns were centrifuged and washed according to the manufacturer’s protocol and the RNA was eluted using 12 μl of RNAse-free water. To determine the concentration and assess the quality, the RNA was measured using Agilent 2100 Bioanalyzer and RNA 6000 Pico Kit (Agilent Technologies, USA).

For RNA extraction from tissues, 45-50 mg samples were cut from tissue specimens preserved in RNAlater (Thermo Fisher Scientific, USA), overlaid with 700 μL QIAzol Lysis Reagent (QIAGEN) in Lysing Matrix A tubes (MP Biomedicals), and homogenized twice for 40 seconds at 6m/s using FastPrep-24™ device (MP Biomedicals). Differential extraction of long- and short- RNA enriched fractions was carried out using miRNeasy mini and micro kits (QIAGEN) according to the manufacturer’s protocol. Long RNA fractions were subjected to on-column treatment with RNase-Free DNase Set (QIAGEN), while short RNA fractions were treated using Ambion^®^ DNA-free™ kit (Thermo Fisher Scientific, USA).

### RNA sequencing

Small RNA libraries were constructed using CleanTag^®^ Small RNA Library Prep Kit (Trilink Biotechnologies, USA) according to the manufacturer’s protocol. Briefly, one-fifth of the total RNA obtained from EVs and 100 ng of tissue RNA was used for library construction. 3’ and 5’ adapters were ligated to the RNA and then the tagged RNA library was reverse transcribed. After that index primers were added, and the RT product was amplified by PCR using 15 cycles for tissue RNA and 18 cycles for EV RNA. The obtained libraries were analyzed with Agilent 2100 Bioanalyzer and Agilent High Sensitivity DNA Chip (Agilent Technologies, USA). The libraries were cleaned using Blue Pippin DNA Size Selection with 3% gel Blue Pippin Cassette (Sage Science, USA), setting a tight target length to 140 bp, thus selecting a size range from 126 – 154 bp. Then the library concertation was measured using Qubit and the libraries were diluted as required and sequenced on Illumina NextSeq500 instrument using NextSeq 500/550 Mid Output Kit v2.5 (150 cycles) (Illumina, USA).

Transcriptome libraries were constructed using MGIEasy RNA Directional Library Prep Kit (MGI, China) according to the manufacturer’s protocol. Briefly, 200 ng of total RNA was subjected to rRNA removal with the MGIEasy rRNA depletion kit (MGI, China). Next, the RNA was fragmented into 250 bp pieces, reverse transcribed and the second strand was synthesized. Then adapters were ligated to the product, and it was amplified by PCR. The length of the inserts was measured using Agilent High Sensitivity DNA Chip on Agilent 2100 Bioanalyzer (Agilent Technologies, USA). The concentration was measured using a Qubit^®^ fluorometer (Thermo Fisher Scientific, USA). The libraries were then pooled according to the index sets, as required by the manufacturer for circularization, circularized, digested, and then sequenced with the MGI DNBSEQ-G400 sequencer (MGI, China).

### Statistical analysis

The obtained raw data in FASTQ format were analyzed using an *ad-hoc* R script pipeline, which included the trimming of adapters using Cutadapt (14), mapping of reads against Ensembl human genome (GRCh38) using Bowtie2 (15), repositioning of multi-aligned reads using ShortStack (16), counting using Rsubread package (17) with GRCh38 and miRbase, GtRNAdb, LNCipedia, lncRNAdb, piRBase, piRNABank, and piRNAdb annotations. To assess the representation of various RNA biotypes in EVs, the reads mapped to overlapping features in human genome were prioritized in the following order: miRNAs > tRNAs > rRNA > mRNAs > pseudogenes > snRNAs > snoRNAs > piRNAs > lncRNAs > miscRNAs. For transcriptome libraries, reads were mapped using STAR (18). For differentially expressed gene (DEG) analysis, the reads were normalized and analyzed using edgeR package. Multiple testing correction was done by the Benjamini-Hochberg procedure and adjusted (adj.) *p*-value of ≤0.05 was considered to be significant.

The receiver operating characteristic (ROC) curve was constructed from the normalized read counts and the area under the curve (AUC) was calculated to evaluate the predictive value of the selected RNA biomarkers. Cutoff points on the ROC curves for determining sensitivity and specificity were defined using Youden Index (19).

## Results

### Dynamics of EV levels in BC patients during the treatment

**Figure 1:**
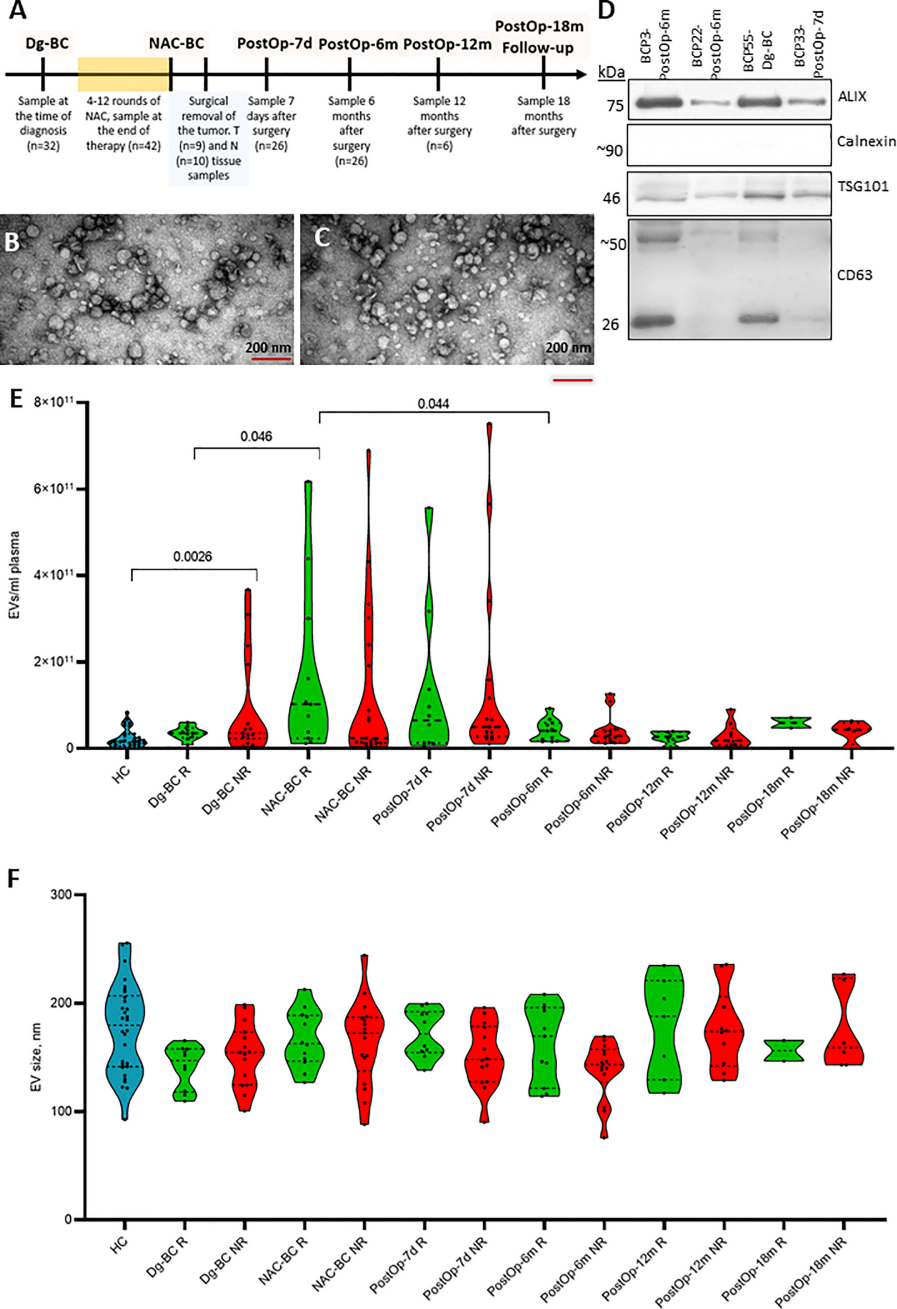
Characterization and quantification of extracellular vesicles. **(A)** The timeline of clinical sample collection and the number of samples used for RNA sequencing analysis at each time point. **(B)** Transmission electron microscopy image of EVs isolated from BC patient’s plasma at the time of diagnosis and **(C)** 7 days after surgery. The scale bar is 200 nm. **(D)** Western blot analysis of EV markers (ALIX, TSG101, and CD63) and endoplasmic reticulum protein Calnexin as a negative control. **(E)** Quantification of EVs isolated from plasma of healthy controls (HC) and BC patients at various time points. Patients were dichotomized based on the Miller-Payne grades: patients with grades 1 to 3 were classified as non-responders (NR) and patients with grades 4-5 as responders (R). Statistical significance was assessed using the Wilcoxon test and *p*<0.05 was considered to be significant. **(F)** Particle size of EVs isolated isolated from plasma of HCs and BC patients at various time points measured by NTA. Dg-BC, time of the diagnosis; NAC-BC, end of neoadjuvant chemotherapy; PostOp-7d, 7 days after breast surgery; PostOp-6m, 6 months after breast surgery; PostOp-12m, 12 months after breast surgery; PostOp-18m, 18 months after breast surgery.

EVs were isolated from serial plasma samples collected at the diagnosis (Dg-BC), at the end of NAC (NAC-BC) and 7 days (PostOp-7d), 6 (PostOp-6m), 12 (PostOp-12m), and 18 months (PostOp-18m) after the surgery from 32 patients with locally advanced BC and 30 age-matched cancer-free healthy controls (HC) using SEC (**Figure 1A**). To assess the purity, EVs obtained from 4 BC patients were characterized by TEM and WB analysis, whereas all EV preparations were routinely measured by NTA. TEM revealed that both samples collected before and after surgery contain a similar mixture of vesicles ranging in size from 50 to 300 nm, including some vesicles with cup-shape morphology that is typically observed for exosomes using this TEM protocol (**Figures 1B, C**). However, some smaller particles (<35 nm) were also present. Hence, these results suggest that the EV preparations contain a mixture of exosomes and microvesicles and a minor contamination with lipoprotein particles or other non-vesicular extracellular particles such as exomeres. WB results showed that EVs were positive for typical EV markers ALIX, CD63, and TSG101 (**Figure 1D**). The molecular weight of ALIX is ~75 kDa that corresponds to the C-terminal proteolytic cleavage product (20), whereas multiple bands ranging from ~25kDa to ~70kDa detected with anti-CD63 antibody correspond to CD63 core protein and its multiple N-glycosylated forms (21). EVs were negative for calnexin, an endoplasmic reticulum protein, thus showing that the EV preparations do not contain significant contamination of ER membranes. NTA showed that the number of circulating EVs in BC patients ranged from 1.71 x 10^9^ to 7.92 x 10^11^ EVs per ml of plasma (**Figure 1E**). Statistically significant differences in the EV levels between responders and non-responders were not observed, though the number of EVs per ml of plasma was significantly higher in non-responders than in healthy controls. Furthermore, the EV levels significantly increased during the NAC both in responders and non-responders. The EV levels were still high 7 days after the surgery but decresed to the same levels as in cancer-free controls 6 months after the surgery and stayed relatively stable at least till the month 18. As we had not collected a blood sample on the day before surgery, our data does not allow to conclude whether the NAC-induced EV levels remained high 7 days after the surgery or the surgical intervention re-induced the release of EVs. No significant differeces in the EV size range were found between the groups of samples (**Figure 1F**).

### Composition of EV RNA content

On average a total of 4.2 million raw reads were obtained per EV sample, and an average of 2.4 million reads remained after quality control, adaptor trimming, and filtering out fragments smaller than 15 nt, and an average of 61% of reads were mapped to the human genome version GRCh38.

**Figure 2:**
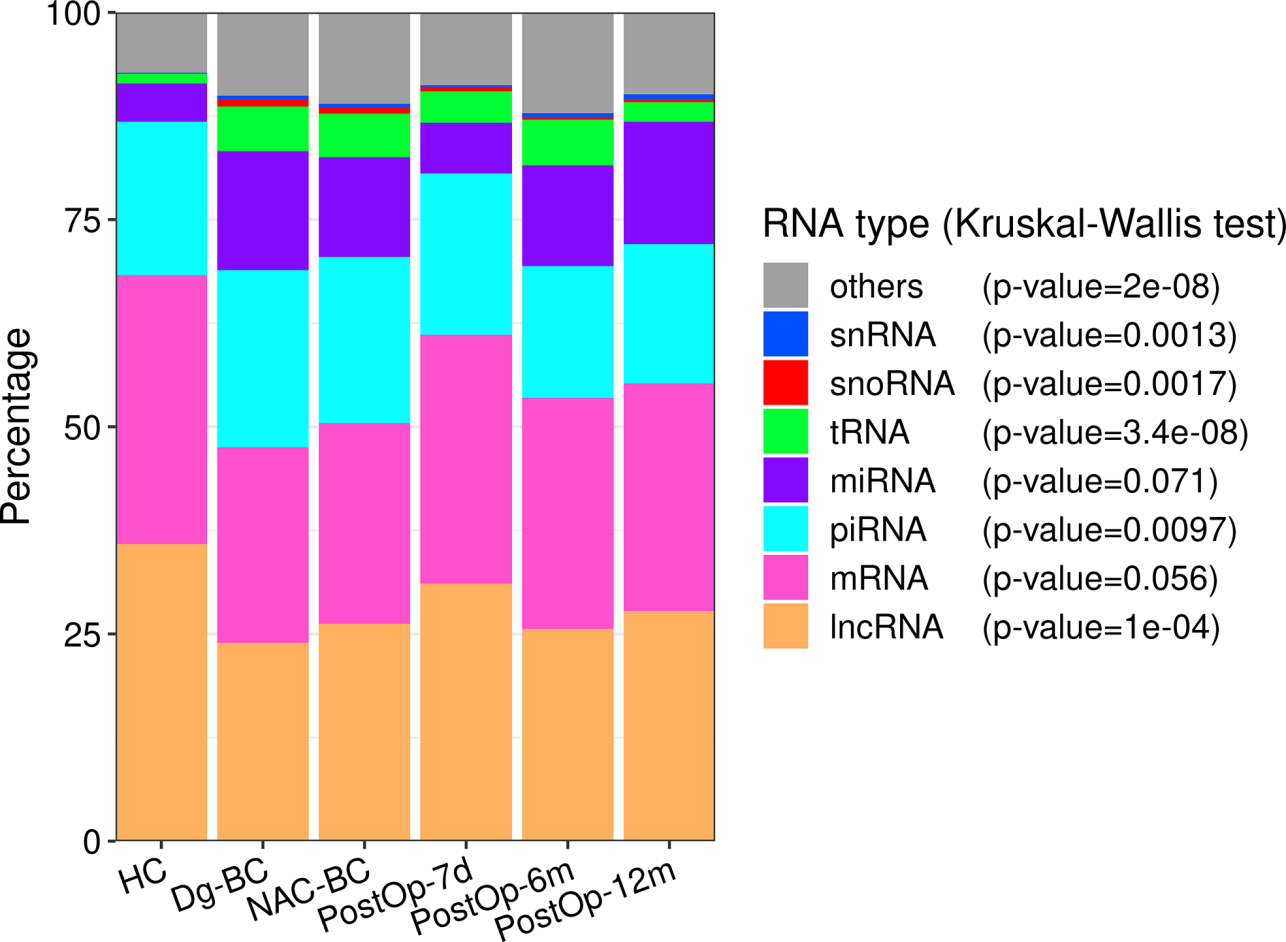
The distribution of RNA biotypes in plasma EVs collected from healthy controls and BC patients at various time points. The statistical significance was determined using the Kruskal-Wallis test and p-value <0.05 was considered statistically significant. HC, healthy controls; Dg-BC, time of the diagnosis; NAC-BC, end of neoadjuvant chemotherapy; PostOp-7d, 7 days after breast surgery; PostOp-6m, 6 months after breast surgery; PostOp-12m, 12 months after breast surgery.

The most abundant RNA biotype in EVs was lncRNA (26%), followed by mRNA (25%), piRNA (18%), miRNA (17%), and tRFs (4%) (**Figure 2**). The fractions of tRFs, snoRNAs, snRNAs, and piRNAs were higher, whereas the lncRNA fraction was lower in BC patients at the time of diagnosis as in HCs. In the subsequent three time points, the fractions of RNA biotypes stayed relatively invariable, whereas 12 months after the surgery, lncRNA, piRNA, and tRF fractions tended to return to levels found in HCs.

### Identification of RNA biomarkers for the prediction of response to NAC

To identify EV-enclosed RNA biomarkers that can predict patients’ response to the NAC at the time of diagnosis, differential expression analysis between responders and non-responders was carried out for each of the major biotypes at two time points: diagnosis and the end of NAC. RNAs with Log2FC>1 and adj. *p*<0.05 were considered to be differentially expressed. Although NAC is expected to elicit EV release from various tissues, we reasoned that the RNA biomarkers that are associated with the presence of a drug-resistant tumor should remain detectable throughout the NAC, therefore only those RNAs that were differentially expressed in both time points were considered as biomarker candidates. Next, the levels of selected candidate biomarkers were compared to HCs and between responders *vs* non-responders at all subsequent time points as well as between tumor and adjacent normal breast tissues. Small RNAs (miRNAs, snoRNAs, snRNAs, piRNAs, tRFs) were analyzed in small RNA libraries constructed from the tumor and normal breast tissues, whereas mRNAs and lncRNAs were analyzed in full transcriptome libraries.

#### miRNAs

**Figure 3:**
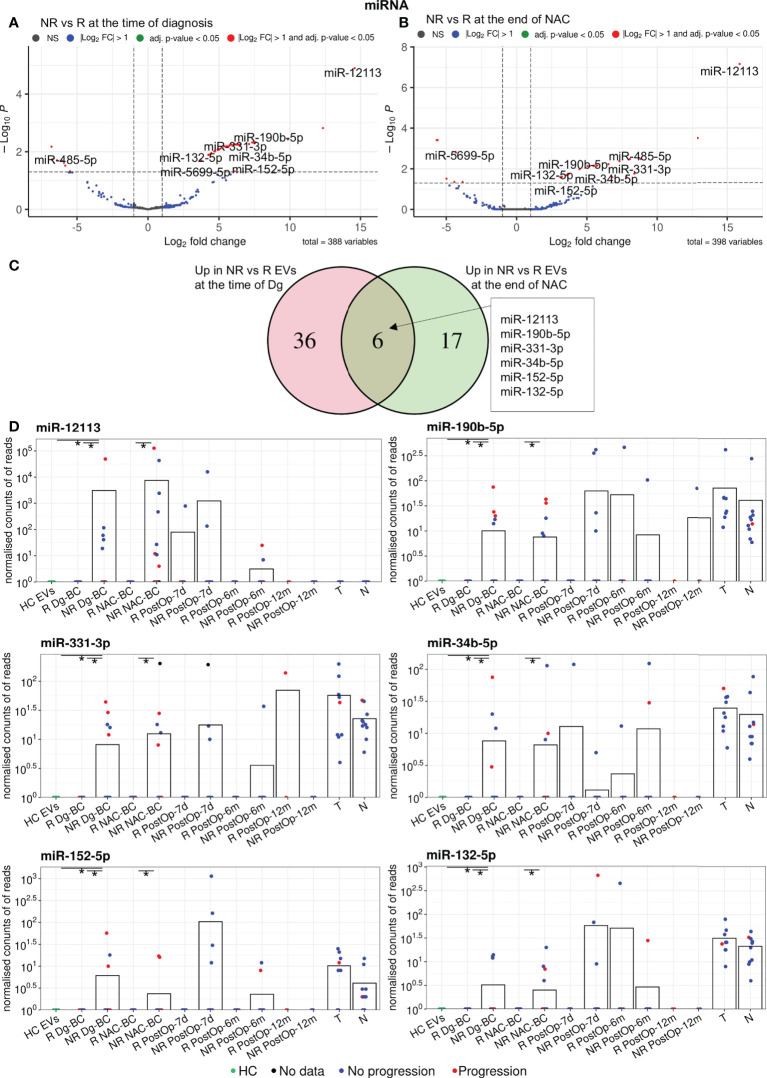
Analysis of differentially expressed miRNAs between non-responders (NRs) and responders (Rs). **(A)** Volcano plot showing the DEGs between NRs and Rs at the time of diagnosis. **(B)** Volcano plot showing the DEGs between NRs and Rs after neoadjuvant chemotherapy (NAC). **(C)** Venn diagram showing the overlap of the DEGs at the time of diagnosis and after NAC. **(D)** Dot plots showing the changes in the EV levels of the selected miRNAs over the course of the disease. Bars represent the mean miRNA levels. Statistical significance was assessed using the Wilcoxon test and *p*<0.05 was considered to be significant. * indicates *p*<0.05. NR, non-responders; R, responders; NAC, neoadjuvant chemotherapy; HC, healthy controls; Dg-BC, time of the diagnosis; NAC-BC, end of neoadjuvant chemotherapy; PostOp-7d, 7 days after breast surgery; PostOp-6m, 6 months after breast surgery; PostOp-12m, 12 months after breast surgery; T, tumor tissue samples; N, normal tissue samples.

Differential expression analysis of miRNAs in responders *vs* non-responders at the time of diagnosis revealed 48 differentially expressed genes (DEGs), including 42 that had higher levels in non-responders (**Figure 3A**). At the end of NAC, 29 DEGs, including 23 miRNAs with higher levels in non-responders were found (**Figure 3B**). Six of the non-responder-associated DEGs coincided at both time points and were selected as biomarker candidates (**Figure 3C**; **Table 2**). **Figure 3D** shows the changes in their EV levels throughout the course of the disease as well as their levels in BC and normal breast tissues. They could distinguish responders from non-responders with high specificity (Sp=1) but low sensitivity (Sn=0.18-0.31) as they were detectable only in a fraction of non-responders. Importantly, none of these 6 miRNAs was detectable in HC EVs. Moreover, miR-190b-5p and miR-331-3p were more frequently detected in patients who experienced disease progression within 18 months after the surgery than in those, who stayed disease-free, yet the difference did not reach statistical significance. After the surgery, the levels of miR-12113 and miR-34b-5p decreased in the majority but not all patients, whereas miR-190b-5p, miR331-3p, miR-152-5p, and miR-132-5p did not significantly decrease in the post-operation samples. All of these miRNAs, except miR-12113, were detectable in BC and normal breast tissues, but none of them was significantly overexpressed in cancer. Taken together, these data suggest that tumor tissue is one but not the only source of these miRNAs in the bloodstream.

**Table 2:** Differentially expressed genes in non-responders *vs* responders at the time of diagnosis and at the end of NAC.

Gene	NR vs R, Dg-BC					NR vs R, NAC-BC			Function
	Log2FC	Adjusted P-value	AUC	Sensitivity	Specificity		Log2FC	Adjusted P-value	
**miRNA**									
**miR-12113**	14.591	1.2E-5	0.66	0.31	1		15.878	6.96E-8	*Unknown*
**miR-190b-5p**	6.200	0.006	0.66	0.31	1		5.742	0.007	Highest upregulated miRNA in ER+ BC, high expression of miR-190b was associated with a prolonged metastasis free survival independently to ER status and treatment as well as a prolonged event-free survival (22). Has been reported both as tumor suppressor and oncogene in multiple cancers (23).
**miR-331-3p**	5.858	0.006	0.66	0.31	1		6.536	0.006	Overexpressed in metastatic BC (24), high expression is associated with worse prognosis in BC (25). Found in PC-CAF EVs, promotes tumor growth (26)
**miR-34b-5p**	5.755	0.006	0.66	0.25	1		5.520	0.007	Shows anti-tumorigenesis role in breast cancer cells by targeting ARHGAP1 (27), upregulated in response to anti-cancer treatment in mice (28)
**miR-152-5p**	5.392	0.007	0.59	0.18	1		3.559	0.020	miR-152-5p suppresses the progression of glioma (29). Overexpression in LC inhibits cell viability, promotes apoptosis, and reduces migration and invasion (30)
**miR-132-5p**	4.248	0.013	0.59	0.19	1		3.720	0.018	Targets the TGFb, the Wnt and the MAP kinase pathways, higher expression in osteosarcoma patients that do not respond to therapy (31)
**lncRNA**									
**lnc-PARP8-6**	10.049	0.030	0.63	0.25	1		10.089	0.015	Unknown
**lnc-DPH7-1**	8.653	0.037	0.63	0.25	1		8.461	0.019	Unknown
**lnc-KLF17-1**	8.494	0.037	0.63	0.25	1		7.389	0.019	Unknown
**lnc-ALX1-2**	8.839	0.037	0.59	0.19	1		10.538	0.015	Unknown
**snoRNA**									
**SNORD111**	7.331	0.005	0.59	0.19	1		3.794	0.020	Unknown

ER+, estrogen receptor-positive; BC, breast cancer; PC, pancreatic cancer; CAF, cancer-associated fibroblast; EVs, extracellular vesicles; LC, liver cancer.

#### lncRNAs

**Figure 4:**
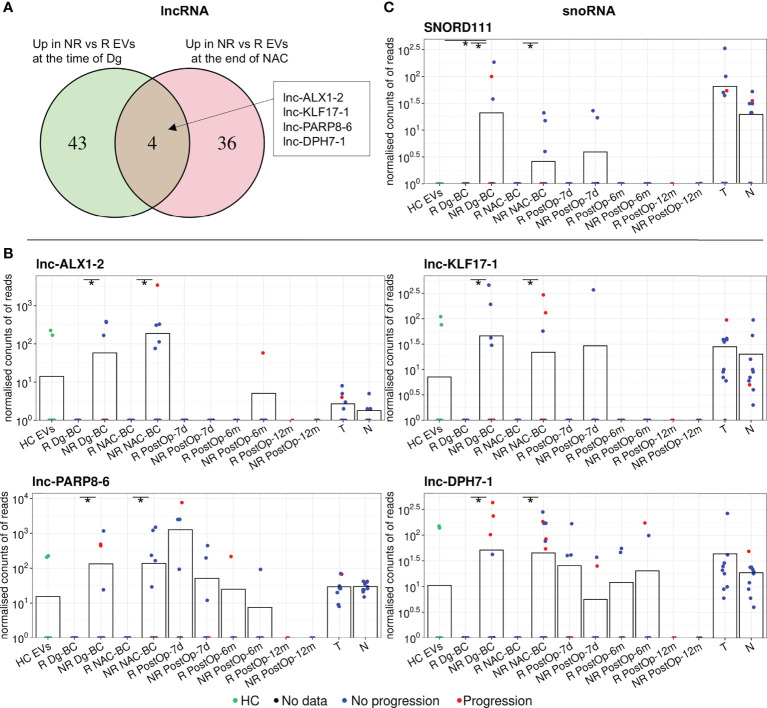
Analysis of other differentially expressed non-coding RNA biotypes between non-responders (NRs) and responders (Rs). **(A)** Venn diagram showing the overlap of differentially expressed lncRNAs between NRs and Rs at the time of diagnosis and after neoadjuvant chemotherapy (NAC). **(B)** Dot plots showing the changes in the EV levels of the selected lncRNAs over the course of the disease. **(C)** Dot plots showing the changes in the EV levels of SNORD111 over the course of the disease. Bars represent the mean RNA levels. NR, non-responders; R, responders; NAC, neoadjuvant chemotherapy; HC, healthy controls; Dg-BC, time of the diagnosis; NAC-BC, end of neoadjuvant chemotherapy; PostOp-7d, 7 days after breast surgery; PostOp-6m, 6 months after breast surgery; PostOp-12m, 12 months after breast surgery; T, tumor tissue samples; N, normal tissue samples. * indicates p<0.05.

Differential expression analysis of lncRNAs revealed 42 DEGs between responders and non-responders (40 of them higher in non-responders) at the time of diagnosis and 49 DEGs after the NAC (47 of them higher in non-responders), but only 4 of them - lnc-ALX1-2, lnc-KLF17-1, lnc-DPH7-1, and lnc-PARP8-6, coincided in both time points (**Figure 4A**; **Table 2**). Levels of these lncRNAs in EVs and tumor and normal breast tissues are shown in **Figure 4B**. The levels of lnc-ALX1-2 and lnc-KLF17-1 distinguished responders from non-responders with high specificity and dropped after the surgical removal of the tumor, nevertheless, they were also detectable in 2 cancer-free controls (6.67%) suggesting that the release of these RNAs in plasma EVs is not always associated with the presence of cancer. Lnc-PARP8-6 and lnc-DPH7-1 also distinguished responders from non-responders in the pre-operation samples, yet their levels increased after the surgery both in responders and non-responders, suggesting that their release is related to the tissue damage and/or wound healing.

#### mRNAs

A comparison of mRNA profiles in responders and non-responders at the time of diagnosis revealed only one differentially expressed gene – G kinase anchoring protein 1 (GKAP1) that had a significantly higher level (Log2FC 12.86; adj.*p* = 0.02) in non-responder EVs. However, GKAP1 mRNA was also present in 20% of HCs and increased in responders during the NAC which limits its value as a predictive biomarker.

#### Other noncoding RNAs

Analysis of other small noncoding RNA biotypes revealed 16 differentially expressed snoRNAs, 6 snRNAs and 6 tRFs, whereas no DEGs were found among piRNAs. However, only one of them - SNORD111 remained differentially expressed after the NAC. SNORD111 is not detectable in HC EVs and shows high specificity but low sensitivity (Sn=0.19) for predicting response to NAC (**Figure 4C**; **Table 2**).

### NAC-induced RNAs

**Figure 5:**
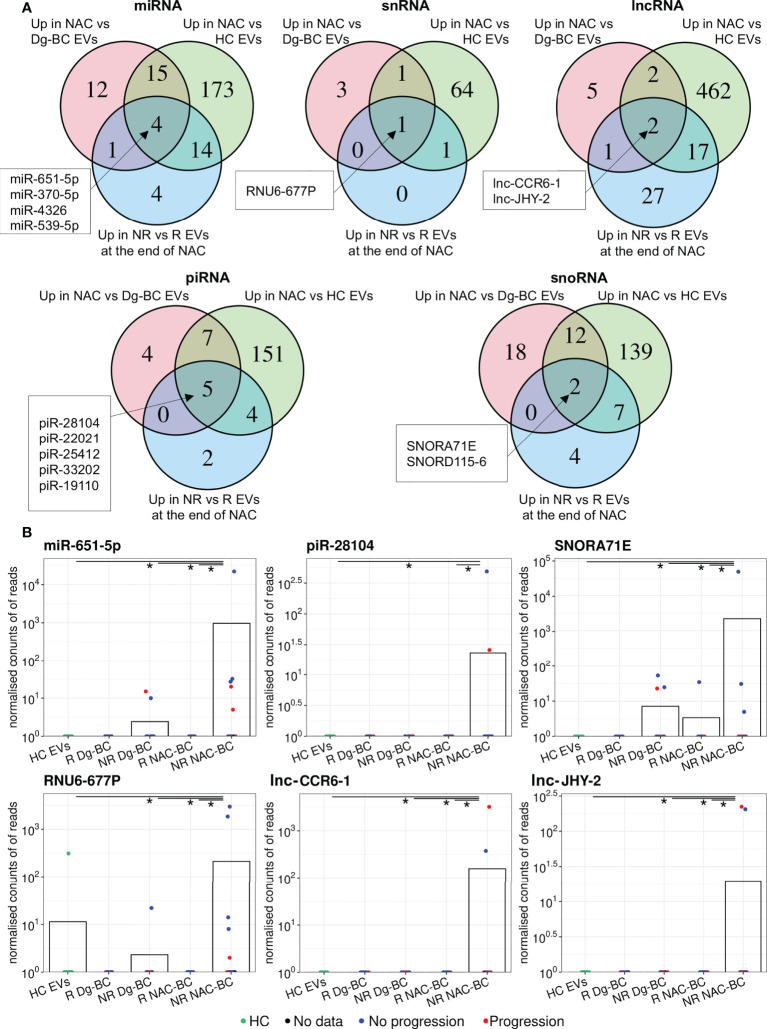
Analysis of NAC-induced EV RNAs. **(A)** Venn diagrams showing the overlap of RNAs upregulated at end of NAC *vs* time of diagnosis; upregulated in BC patients at the end of NAC *vs* healthy controls (HCs) and upregulated in non-responders(NRs) *vs* responders(Rs) at the end of NAC. **(B)** Dot plots showing the changes in the EV levels of the selected NAC-induced RNAs over the course of the disease. Bars represent the mean RNA levels. NR, non-responders; R, responders; NAC, neoadjuvant chemotherapy; HC, healthy controls; Dg-BC, time of the diagnosis; NAC-BC, end of neoadjuvant chemotherapy;Up, upregulated.

To identify RNAs that are induced by the NAC and potentially contribute to drug resistance or disease progression, we performed the following differential expression analyses (1): NAC-BC *vs* Dg-BC to identify NAC-induced RNAs (2); NAC-BC *vs* HCs to identify RNAs that are specific to BC and (3) non-responders *vs* responders (or patients with or without progression) at the end of NAC to identify RNAs that are associated with poor response to the NAC or clinical progression within 18 months after the surgery. Five piRNAs: piR-28104, piR-22021, piR-25412, piR-33202, and piR-19110, 4 miRNAs: miR-651-5p, miR-370-5p, miR-4326, and miR-539-5p, 2 lncRNAs: lnc-CCR6-1 and lnc-JHY-2, snoRNAs: SNORA71E and SNORD115-6 and 1 snRNA: RNU6-677P were found to be elicited by the NAC, present in BC EVs at the end of NAC at significantly higher levels than in HCs and have higher levels in non-responders than responders (**Figure 5A**; **Table 3**). In addition, two snoRNAs: SNORD28 and SNORD115-5, and one piRNA: piR-33202 were found, when the differential expression analysis between patients with and without progression was carried out. Of note, piR-33202 was common in both analyses. **Figure 5B** shows the EV levels of RNAs that were the most significantly induced by NAC.

**Table 3:** NAC-induced RNAs.

Gene	NAC-BC vs Dg-BC		NAC-BC vs HC		NR vs R in NAC-BC		P vs NP in NAC-BC	
	Log2FC	Adjusted P-value	Log2FC	Adjusted P-value	Log2FC	Adjusted P-value	Log2FC	Adjusted P-value
**piRNA**								
piR-28104	6.622	0.0001	6.622	2.12E-05	7.484	0.034	*ns*	*ns*
piR-22021	5.467	0.0001	5.46721	2.24E-05	6.321	0.034	*ns*	*ns*
piR-25412/piR-2975/piR-2974/piR-26842/piR-3025/piR-31225	5.973	0.0005	7.557871	1.57E-05	8.423	0.034	*ns*	*ns*
piR-33202	4.002	0.002	5.00222	2.17E-05	5.824	0.034	5.319	0.0099
piR-19110	3.793	0.0387	10.28104	1.29E-06	8.939	0.018	*ns*	*ns*
**miRNA**								
miR-651-5p	9.294	1.65E-05	12.04896	3.38E-07	12.917	0.0003	*ns*	*ns*
miR-370-5p	2.827	0.0002	2.827	3.35E-05	3.601	0.0204	*ns*	*ns*
miR-4326	2.619	0.0002	2.619044	4E-05	3.225	0.0249	*ns*	*ns*
miR-539-5p	2.478	0.0002	2.478192	6.14E-05	3.225	0.0249	*ns*	*ns*
**lncRNA**								
lnc-CCR6-1	9.420	6.73E-05	9.420533	5.05E-06	10.288	0.017	*ns*	*ns*
lnc-JHY-2	6.41	6.73E-05	6.40986	5.65E-06	7.2213	0.020	*ns*	*ns*
**snoRNA**								
SNORA71E	8.360	0.000335	13.2307	3.02E-08	9.746	0.024	*ns*	*ns*
SNORD115-6	5.636	0.005878	11.806	3.02E-08	9.105	0.0369	*ns*	*ns*
SNORD28	6.590	0.001752	15.030	5.79E-09	*ns*	*ns*	6.773	0.009
SNORD115-5	4.260	0.027074	11.558	3.02E-08	*ns*	*ns*	6.517	0.009
**snRNA**								
RNU6-677P	6.269	0.0008	3.466	0.033	10.724	9.13E-05	*ns*	*ns*

NR, non-responder; R, responder; P, progression; NP, no progression; ns, not significant.

## Discussion

EVs have gained increasing attention as a source of cancer-derived biomarkers in liquid biopsies. Potentially, they may have several advantages over other sources like CTCs and cell-free DNA or RNA: they are highly abundant in circulation as compared to CTCs, they protect their molecular cargo against degradation, they carry molecular signatures associated with specific phenotypes of parental cells, and transfer phenotypic traits to their recipient cells (12, 32). In the current study, we investigated the dynamics of EV levels and their RNA cargo at various time points during the treatment of BC patients and correlated the changes in their RNA content with clinical events.

Elevated levels of plasma EVs have been found in patients with various solid tumors (33–35), including BC (36). A study by König et al, 2018 showed that the EV concentration increased during NAC and high pre-treatment EV concentration was associated with therapy failure in BC patients (36). In line with this study, we found that the pre-treatment EV levels in non-responders were significantly higher than in healthy controls and were slightly higher than in responders. The EV concentration increased significantly both in responders and non-responders during the NAC, stayed at approximately the same level on day 7 after surgery and decreased to the level found in HCs by 6 months after surgery. However, the tissue and cellular source of the excess EVs remained unknown. Several studies have shown that the treatment of breast cancer cells with chemotherapeutic drugs induces EV secretion (37, 38). However, at least in animal experiments, the half-life of EVs in the bloodstream was estimated to be less than an hour (39). If the excess EVs were derived predominantly from cancer tissues, it could be expected that they are cleared from the circulation within a few days. Moreover, elevated EV concentration has been found in a variety of other diseases (12), as well as in healthy individuals during exercise (40, 41) or pregnancy (42, 43). Therefore, the secretion of EVs appears to be a common feature of various diseases and physiological conditions that is triggered by tissue damage and various stress factors. Conceivably, in BC patients undergoing NAC, the excess EVs are derived from the tumor, liver, blood cells and other cell types responding to the presence of cancer, tissue damage or chemotherapeutic drugs.

Next, we studied whether the RNA cargo of EVs could inform about patient’s response to the NAC. We found that the changes in the EV levels were associated with the alterations in the proportions of various RNA biotypes with tRFs, lncRNAs and snoRNAs being the most significantly shifted. Analysis of EV RNA content revealed 6 miRNAs, 4 lncRNAs, and 1 snoRNA that had significantly higher levels in EVs from non-responders than responders at the time of diagnosis and throughout the NAC. Importantly, they were not detectable or had significantly lower levels in EVs from cancer-free controls thus making them attractive biomarker candidates for the prediction of a patient’s response to chemotherapy at the time of diagnosis. All of the identified biomarker candidates had specificity of 1 for discriminating between responders and non-responders, but the sensitivity was ranging from 0.18 to 0.31, suggesting that individually they have moderate clinical value, whereas combining them in a biomarker panel would increase their translatablity. However, in the current cohort of patients, 6 out of the 20 non-responders were negative for all of the biomarker candidates thus showing that this biomarker panel would not be suitable for predicting response to the NAC in a subgroup of patients. Noteworthly, this subgroup was not associated with the hormone receptor expression or HER2 status. Hence, larger cohort of patients would be needed to discover biomarkers in various subgroups of BC.

Increasing evidence suggests that in some patients chemotherapeutic drugs used in the NAC setting can induce metastatic progression of the disease (7, 44). Several recent studies have demonstrated that cytotoxic drugs that are broadly used in NAC regimens elicit EV release from cancer cells and chemotherapy-induced EVs facilitate the formation of pre-metastatic niche, accelerate metastasis, stemness, and chemoresistance of BC cells (37, 38, 45). Therefore, we searched for EV RNAs that were induced by NAC and may contribute to the chemoresistance or progression of BC and that resulted in the identification of 14 RNAs representing piRNA, miRNA, lncRNA, snoRNA, and snRNA biotypes. In addition, 1 piRNA and 2 snoRNAs were identified in patients with the early progression of the disease.

Among the resistance-associated miRNAs was miR-190b-5p, which previously has been found to be strongly overexpressed in ER-positive vs ER-negative BCs (22). A high expression level of miR-190b-5p in BC tissues has been associated with poor survival and shown to promote proliferation and migration of BC cells (46). MiR-331-3p has been found to be overexpressed in metastatic BC (24) and a high expression level in BC tissues has been associated with poor prognosis in BC (25), whereas a high expression level of miR-132-5p was associated with the resistance to ifosfamide in osteosarcoma patients (31).

Among the NAC-induced miRNAs in non-responders is miR-539-5p, which has been shown to act as a tumor suppressor in BC by targeting EGFR, LAMA4, and SP1 thus leading to the inhibition of proliferation and migration of BC cells (47–49). Thus one intriguing possibility is that BC cells are actively sorting miR-539-5p into EVs to deplete its intracellular concentration, which in turn leads to the more aggressive behavior of cancer cells in non-responders, similarly as demonstrated in prostate cancer cells treated with fludarabine (50). Alternatively, miR-539-5p has also been implicated in anti-inflammatory response (51), hence it is possible that its induction by NAC is related to the inflammatory response to tissue damage induced by NAC.

A study by Yang et al. had previously demonstrated that NAC elicits secretion of miR-378a-3p and miR-378d enriched EVs from BC cells that in turn activate WNT and NOTCH stem cell pathways in drug-naïve BC cells leading to the acquisition of drug resistance (45). We also detected these miRNAs in EVs from BC patients, though we did not observe significant induction by NAC.

lncRNAs are non-coding transcripts that range from 200 nucleotides up to ~100 kilobases in length and affect many biological and pathological processes by regulating gene expression at transcriptional and post-transcriptional levels (52). An increasing number of studies reveal crucial roles of lncRNAs in the proliferation, invasion, drug resistance, and metastasis of BC, and several previous studies have found fragments of lncRNAs in EVs isolated from BC patients’ blood that are associated with the disease status or prognosis (53). Nevertheless, the functions of the resistance-associated or NAC-induced lncRNAs identified in the current study are unknown.

snoRNAs are 60-300 nucleotides long non-coding RNAs that primarily accumulate in the nucleoli and are responsible for the posttranscriptional modification and maturation of ribosomal RNAs. Recent evidence suggests that snoRNAs also regulate alternative splicing and editing of mRNAs (54). An increasing number of studies show that their expression is altered in various cancers and they contribute to various processes of cancer progression (54). SNORD115 which we identified as a NAC-induced gene in non-responders and patients with early progression, has been previously found as the initial regulator of BC progression (55). However, to the best of our knowledge, snoRNA content in BC EVs has not been previously studied. Given that more than 2000 snoRNA genes are found in the human genome (56), they appear to be a rich, yet an unexplored source of BC biomarkers.

Taken together, this study demonstrated that a substantial fraction of plasma EVs in BC patients are produced due to the disease processes or treatment. The EV RNA cargo consists of various RNA biotypes whose diagnostic and prognostic significance has not been explored before. The EV RNA cargo in BC patients is altered as compared to cancer-free healthy controls and dynamically reflects the clinical events. A set of BC-specific RNA biomarkers that can predict patients’ response to the NAC at the time of diagnosis was identified. Individually, they had very high specificity yet low sensitivity. Another set of RNAs that are induced by NAC in EVs from non-responders or patients with early disease progression was identified and warrants further functional studies.

## Data availability statement

The datasets presented in this study can be found in online repositories. The names of the repository/repositories and accession number(s) can be found below: https://www.ebi.ac.uk/arrayexpress/experiments/E-MTAB-12014/, E-MTAB-12014.

## Ethics statement

All the experimental protocols involving human data were in accordance with the Declaration of Helsinki. The patients were enrolled in the study after the patients’ informed written consent was obtained.The study protocol was approved by the Latvian Central Medical Ethics Committee (decision No. 1839).

## Author contributions

AL, ML, and JE designed research, LS, JA, LK, EE, KE, and IR-S performed research and participated in the analysis and interpretation of the results, and PZ performed the RNAseq data analysis and statistical analyses. KE, EA, and IL-K collected the clinical samples and information. AL and LS wrote the manuscript. EE, ML, and JE revised the manuscript. All authors have read and approved the manuscript.

## Funding

This work was funded by the ERDF project No. 1.1.1.1/18/A/084.

## Acknowledgments

We are thankful to our colleagues from the Genome Database of Latvian Population for providing the plasma samples used in this study.

## Conflict of interest

Author IR-S is employed by Genera Ltd.

The remaining authors declare that the research was conducted in the absence of any commercial or financial relationships that could be construed as a potential conflict of interest.

## Publisher’s note

All claims expressed in this article are solely those of the authors and do not necessarily represent those of their affiliated organizations, or those of the publisher, the editors and the reviewers. Any product that may be evaluated in this article, or claim that may be made by its manufacturer, is not guaranteed or endorsed by the publisher.

## Abbreviations

BC, breast cancer; DEG, differentially expressed gene; EVs, extracellular vesicles; GO, gene ontology; NTA, nanoparticle tracking analysis; TEM, transmission electron microscopy; TNBC, triple-negative breast cancer; Dg-BC, time of the diagnosis; NAC-BC, end of neoadjuvant chemotherapy; PostOp-7d, 7 days after breast surgery; PostOp-6m, 6 months after breast surgery; PostOp-12m, 12 months after breast surgery; PostOp-18m, 18 months after breast surgery; NAC, neoadjuvant chemotherapy; HC, healthy controls; HR, hormone receptor; pCR, pathological complete response; CTCs, circulating tumor cells; lncRNA, long noncoding RNA; miRNA, microRNA; piRNA, piwi-interacting RNA; circRNA, circular RNA; snRNA, small nuclear RNA; snoRNA, small nucleolar RNA; tRF, tRNA-derived small RNA; SEC, size exclusion chromatography; WB, Western blot; NR, non-responder; R, responder.
